# Comparison of perfusion models for quantitative T1 weighted DCE-MRI of rectal cancer

**DOI:** 10.1038/s41598-017-12194-w

**Published:** 2017-09-20

**Authors:** Tanja Gaa, Wiebke Neumann, Sonja Sudarski, Ulrike I. Attenberger, Stefan O. Schönberg, Lothar R. Schad, Frank G. Zöllner

**Affiliations:** 1 Computer Assisted Clinical Medicine, Medical Faculty Mannheim, Heidelberg University, Heidelberg, Germany; 2 Institute of Clinical Radiology and Nuclear Medicine, Medical Faculty Mannheim, Heidelberg University, Heidelberg, Germany

## Abstract

In this work, the two compartment exchange model and two compartment uptake model were applied to obtain quantitative perfusion parameters in rectum carcinoma and the results were compared to those obtained by the deconvolution algorithm. Eighteen patients with newly diagnosed rectal carcinoma underwent 3 T MRI of the pelvis including a T_1_ weighted dynamic contrastenhanced (DCE) protocol before treatment. Mean values for Plasma Flow (PF), Plasma Volume (PV) and Mean Transit Time (MTT) were obtained for all three approaches and visualized in parameter cards. For the two compartment models, Akaike Information Criterion (AIC) and ${{\boldsymbol{\chi }}}^{{\bf{2}}}$ were calculated. Perfusion parameters determined with the compartment models show results in accordance with previous studies focusing on rectal cancer DCE-CT (PF_2CX_ = 68 ± 44 ml/100 ml/min, PF_2CU_ = 55 ± 36 ml/100 ml/min) with similar fit quality (AIC:169 ± 81/179 ± 77, ${{\boldsymbol{\chi }}}^{{\bf{2}}}$:10 ± 12/9 ± 10). Values for PF are overestimated whereas PV and MTT are underestimated compared to results of the deconvolution algorithm. Significant differences were found among all models for perfusion parameters as well as between the AIC and ${{\boldsymbol{\chi }}}^{{\bf{2}}}$ values. Quantitative perfusion parameters are dependent on the chosen tracer kinetic model. According to the obtained parameters, all approaches seem capable of providing quantitative perfusion values in DCE-MRI of rectal cancer.

## Introduction

Tissue perfusion and permeability can be measured non-invasively by dynamic contrast enhanced magnetic resonance imaging (DCE-MRI)^1^. A bolus of paramagnetic contrast media is injected as a tracer for the determination of hemodynamic parameters by analyzing the signal variation with respect to time. This method enables the determination of tumor specific microvascular parameters such as blood flow, blood volume, mean transit time, and the permeability–surface area product^2^. These parameters can then support the differentiation of malignant and benign tumors or the evaluation of tumor response after therapy and also possibly predict therapy outcome. For quantitative analysis of these perfusion parameters, DCE-MRI combined with either semi quantitative analysis^3^, the model free deconvolution algorithm^4^ or the Tofts-model^5^ has been applied in literature. However, these approaches either do not model the structure of the tissue (deconvolution) or are limited by not directly providing values for plasma flow and mean transit time (Toft’s model). In contrast, multi-compartment models can fulfill these requirements^6^. A priori knowledge of the anatomical and physiological structure of the organ or tissue is usually described in a mathematical model using a multi-compartment approach. Regarding patients with rectal carcinoma, morphological and functional MRI have already been used for diagnosis, staging and control of neoadjuvant chemotherapy or radiation therapy^7^. Currently, if a patient is examined with DCE-MRI, the Tofts model is most widely used in clinical routine^8,9^. However, various other perfusion models exist^10^ and no consensus on the pharmacokinetic model is yet reached^11^. The selection of an adequate pharmacokinetic model is crucial to derive the correct perfusion information from the image data to understand microvascular physiology^12^. Currently, there is no systematic method for the identification of the most specific model with regard to clinical usefulness. It is therefore important to compare existing models and assess their quality in terms of describing the DCE-MRI datasets. The aim of our study was to investigate the results of different pharmacokinetic models for quantitative analysis of DCE-MRI of rectal cancer. We compared the fast deconvolution approach^4^ as a model free method and two different compartment models: the two compartment uptake model (2CU)^13^ and the two compartment exchange model (2CX)^14^.

## Material and Methods

### Patients

This prospective single-center study was approved by the Institutional Review Board (Medical Faculty Mannheim, Heidelberg University, Germany, decision number 2013-628N-MA) and written, informed consent of all patients was obtained. All analyses were carried out in accordance with the ethics board approval. Tracing back from patient data to any individual patient is not possible due to full anonymization. 26 patients with newly-diagnosed untreated rectal cancer were enrolled within a period of 2 years (December 2013 to December 2015) and underwent MRI of the pelvis including perfusion sequences covering their tumor. Eight patients had to be excluded from the study due to poor image quality. This resulted in final evaluation of 13 male and 5 female patients with a mean age of 64 ± 10 years. The tumor grade was staged T2 (4 patients), T3 (13 patients) or T4 (1 patient). The MRI data sets can be downloaded from http://dx.doi.org/10.11588/data/NULJJR.

### MRI

DCE-MRI was performed using a 3 T scanner (Magnetom Trio or Magnetom Skyra, Siemens Healthineers, Erlangen, Germany) with a standard spine coil and body coil. To obtain high temporal und spatial resolution, the examination was performed with a 3D time-resolved angiography with stochastic trajectories (TWIST) sequence^15^ with the following imaging parameters: TR/TE/FA = 3.6 ms/1.44 ms/15°, matrix size = 192 × 144, FOV = 260 × 158 mm², slice thickness = 3.6 mm and parallel imaging with a GRAPPA factor of 2. Images were either acquired in axial plane or tilted in direction of the coronal plane to cover the tumor best as possible. A continuously acquisition over a volume of 20 slices for 5 minutes and 50 seconds with a nominal temporal resolution of 5 s per volume resulted in a total of 70 volumes. Right after the 10th volume had been acquired, 3–18 ml (0.1 to 0.15 ml per kg body weight) of a gadolinium-based contrast agent (CA) (Dotarem, Guerbet, France or Gadovist, Schering AG, Germany) were administered intravenously and followed by a 40 ml saline flush. Both solutions were injected with a flow velocity of 1.5 ml/s.

### Perfusion Analysis

Quantitative analysis of the DCE-MRI image data was performed using three different models: a voxel-by-voxel deconvolution approach (DCE-DECON) with modifications of the original algorithm given in ref.^4,16^ for T1 weighted DCE-MRI^17^, a model based two compartment uptake^10,13^ and a model based two compartment exchange model^10,14^. Brief descriptions of them are given in the following. All three models were implemented in an in-house certified OSIRIX plugin (UMMPerfusion version 1.52)^17,18^. A comparison of the obtained perfusion parameters was conducted afterwards.

### Deconvolution Analysis

The deconvolution analysis was conducted using the following equation:^10^

1 $$f=\,\max \,[{C}_{t}(t){\otimes }^{-1}{C}_{a}(t)]$$

Here, *C*_*t*_(*t*) and *C*_*a*_(*t*) are the CA concentrations as a function of time in a region-of-interest (ROI) in the tissue and inside the artery feeding the region of interest (arterial input function), respectively. The perfusion *f* is the maximum value of the tissue impulse response function, i.e. the deconvolution (⊗^−1^) of the two concentration functions. Eq. 1 is solved by singular value decomposition (SVD) using a regularization of 0.15 times the maximal singular value^19^.

### Two compartment exchange model

**Figure 1:**
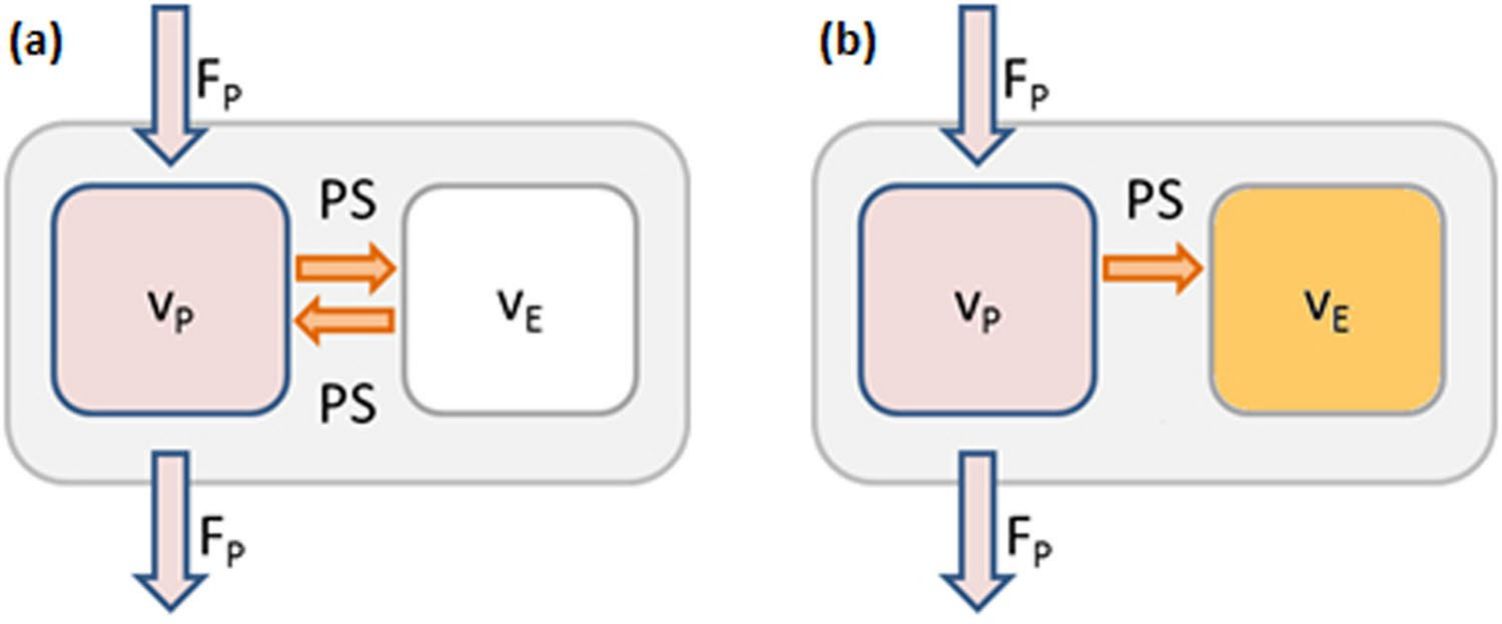
Schematic illustration of (**a**) the two compartment exchange model (2CX) and (**b**) the two compartment uptake model (2CU).

Physiologically, analysis of rectal cancer tumors should consider an exchange of the extracellular contrast agent between arteries and interstitium. In contrast to deconvolution analysis, both two compartment models assume that the tracer in the tissue can distribute in two separate compartments, the plasma space (vascular space) and the extracellular extravascular space (EES). The two compartment exchange model (2CX) is the most general two compartment model that describes the plasma and the interstitial space as two separate compartments with volumes *v*_*p*_ and *v*_*e*_, respectively. Additional to a single compartment model, influx and outflux from the interstitium is taken into account (Fig. 1a).

The concentration of the CA in the tissue is described as

2 $${C}_{t}(t)={v}_{p}{C}_{p}(t)+{v}_{e}{C}_{e}(t)$$

where *C*_*p*_(*t*), the CA concentration in the intravascular plasma, is defined as

3 $$\frac{d{C}_{p}}{dt}=\frac{PS}{{v}_{p}}({C}_{e}(t)-{C}_{p}(t))+\frac{{F}_{p}}{{v}_{p}}({C}_{a}(t)-{C}_{p}(t))$$

and *C*_*e*_(*t*), the CA concentration in the EES space, by

4 $$\frac{d{C}_{e}}{dt}=\frac{PS}{{v}_{e}}({C}_{p}(t)-{C}_{e}(t))$$

*C*_*a*_(*t*) is the CA concentration in the arterial plasma. The permeability *PS* describes the in- and outflow rate of the exchange of the CA between the two compartments *v*_*p*_, the intravascular plasma volume and *v*_*e*_, the EES volume.

Solving Eqs. (3) and (4) yields the bi-exponential tissue response function *H*_2*cx*_ with function parameters *F*_*p*_, *v*_*p*_, *PS*, *v*_*e*_.

Combined with

5 $${C}_{t}(t)={F}_{p}{H}_{2CX}(t)\otimes {C}_{a}(t)$$

the four parameters *F*_*p*_, *v*_*p*_, *PS* and *v*_*e*_ can be determined (for further details ref. to^11^).

### Two compartment uptake model

The two compartment uptake model (2CU) is a simplification of the 2CX: If the mean transit time (MTT) is long compared to measurement time, efflux is negligible from EES. Eq. 4 subsequently simplifies to

6 $$\frac{d{C}_{e}}{dt}=\frac{PS}{{v}_{e}}({C}_{p}(t))$$

and results in a monoexponential tissue response function *H*_2*CU*_(*t*). Solving Eq. 5 with the new tissue response function yields to a 3 parameter model with parameters *PS*, *F*_*p*_ and *v*_*p*_. Compared to the 2CX model the number of model parameters is reduced which means *v*_*e*_ is not accessible.

In other publications, the parameters blood flow and blood volume are used instead of plasma flow and (plasma) volume. A translation between both conventions can easily be performed by a scaling factor based on the haematocrit: *F*_*p*_ = (1−*HCT*)*F*_*B*_, here with a haematocrit value of *HCT* = 0.45.

All models were implemented in an OsiriX plugin (UMMPerfusion, Version 1.5.2)^18^. The plugin allows for executing several DCE-MRI perfusion models simultaneously using the same ROIs during calculations. This is beneficial since the placement of the arterial input function (AIF) and tumor tissue ROIs is crucial for a proper comparison of the models^20^. For compartment models, the plugin also enables an estimation of the Akaike information criterion (AIC) and the chi square (${{\boldsymbol{\chi }}}^{2}$) of the fits assessing the goodness of fit as a parameter for model comparison^21^.

**Figure 2:**
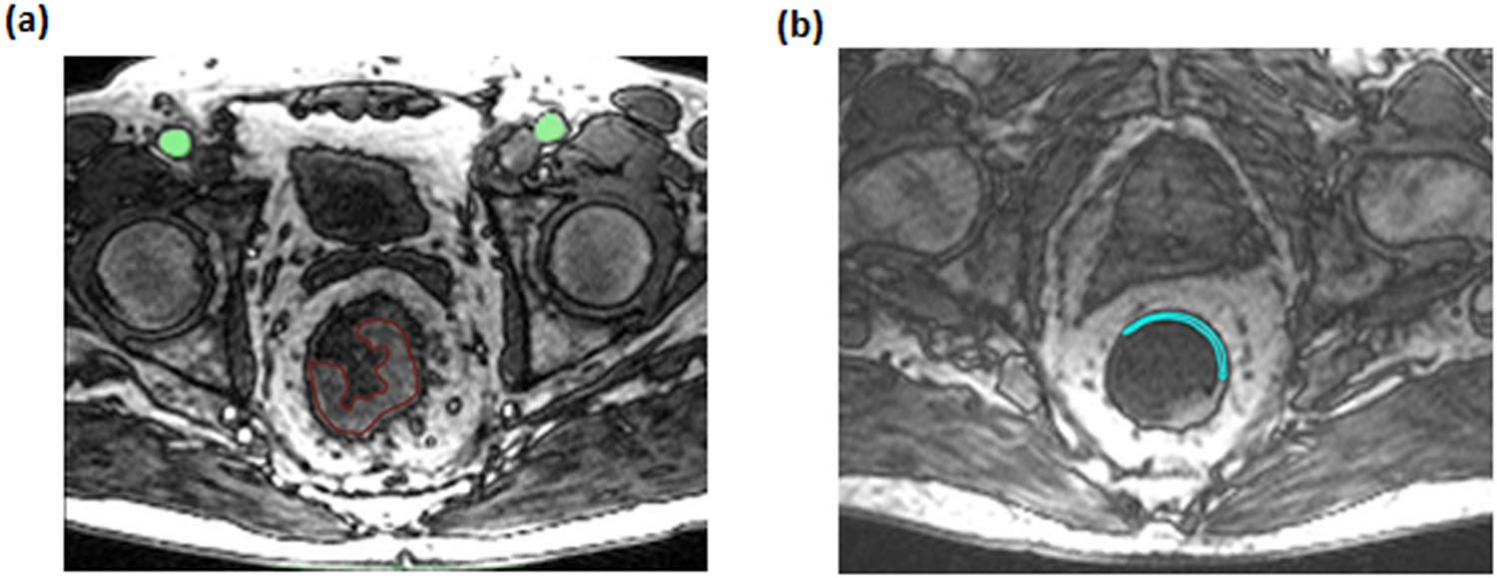
(**a**) AIF and tumor were identified on the slices with their best representation. The AIF (green) was determined by a region growing algorithm with manually set thresholds. Region-of-interest (ROI) drawing was manually performed in consensus with a radiologist. The tumor ROI (red) and healthy rectum wall (**b**) were manually outlined.

In our study, the AIF was determined by carefully placing a ROI in the arteria iliaca externa to avoid inflow effects and to minimize partial volume effects due to the small vessel diameter. It was determined by a region growing algorithm with manually set thresholds. The AIF can differ due to physiological disparities of the left and right artery, for example as a consequence of stenosis^22^. As an alternative to selecting one AIF, we averaged over all voxels of both AIFs ROIs and thus used the signal of the united regions to account for both arteries^23^. During evaluation of measured DCE-MRI data, the area of the AIF and the tumor volume, defined in the slice with maximal volume found, was marked (see Fig. 2a). Analysis of DCE-MRI data was performed for all patients were an AIF could be placed in the left as well as the right side iliac artery. To compare with, a ROI was placed in healthy rectum tissue of 10 patients to obtain mean perfusion parameters (see Fig. 2b).

All data were normalized by subtracting the mean intensity of 5 baseline volumes and a linear relationship of the contrast agent concentration to the measured signal intensities was assumed^24^. The tumor volume was chosen on the slice with the largest tumor intersection and the whole volume was outlined under supervision of a radiologist with three years of experience in abdominal imaging using OsiriX (Version 5.6, Pixmeo Inc., Genève, Switzerland). The calculated values for Plasma Flow (PF), Plasma Volume (PV) and MTT were compared for all three models.

### Statistical Analysis

Statistical analysis was performed using Matlab 2015 (Version 8.1.0.604, the Mathworks, Nattick, MA, USA). Bland-Altman plots were generated to analyze perfusion parameters for every single patient for all possible pairs of two different models, respectively. Lilliefors test was conducted to test for normal distribution within the three groups. Since not all data was normally distributed, nonparametric paired Wilcoxon sign rank test was employed for further analysis of the quantitative perfusion values within different models. A significance level of P < 0.05 was set.

## Results

### Perfusion data analysis

**Figure 3:**
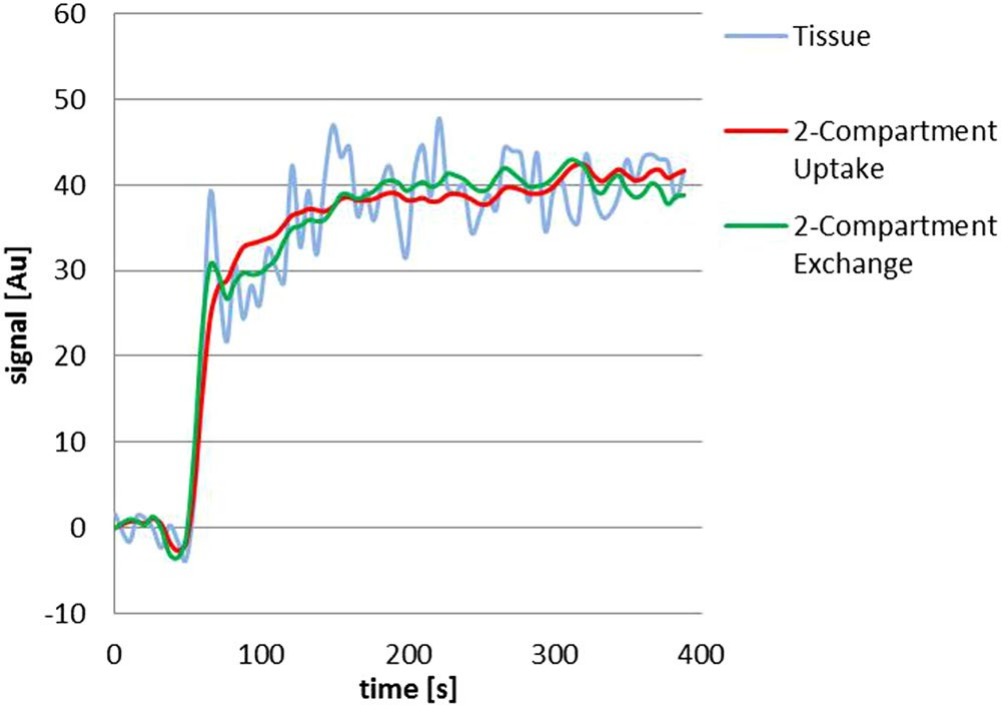
Tumor-tissue signal (blue) over time and fit with 2CU (red) and 2CX (green).

**Figure 4:**
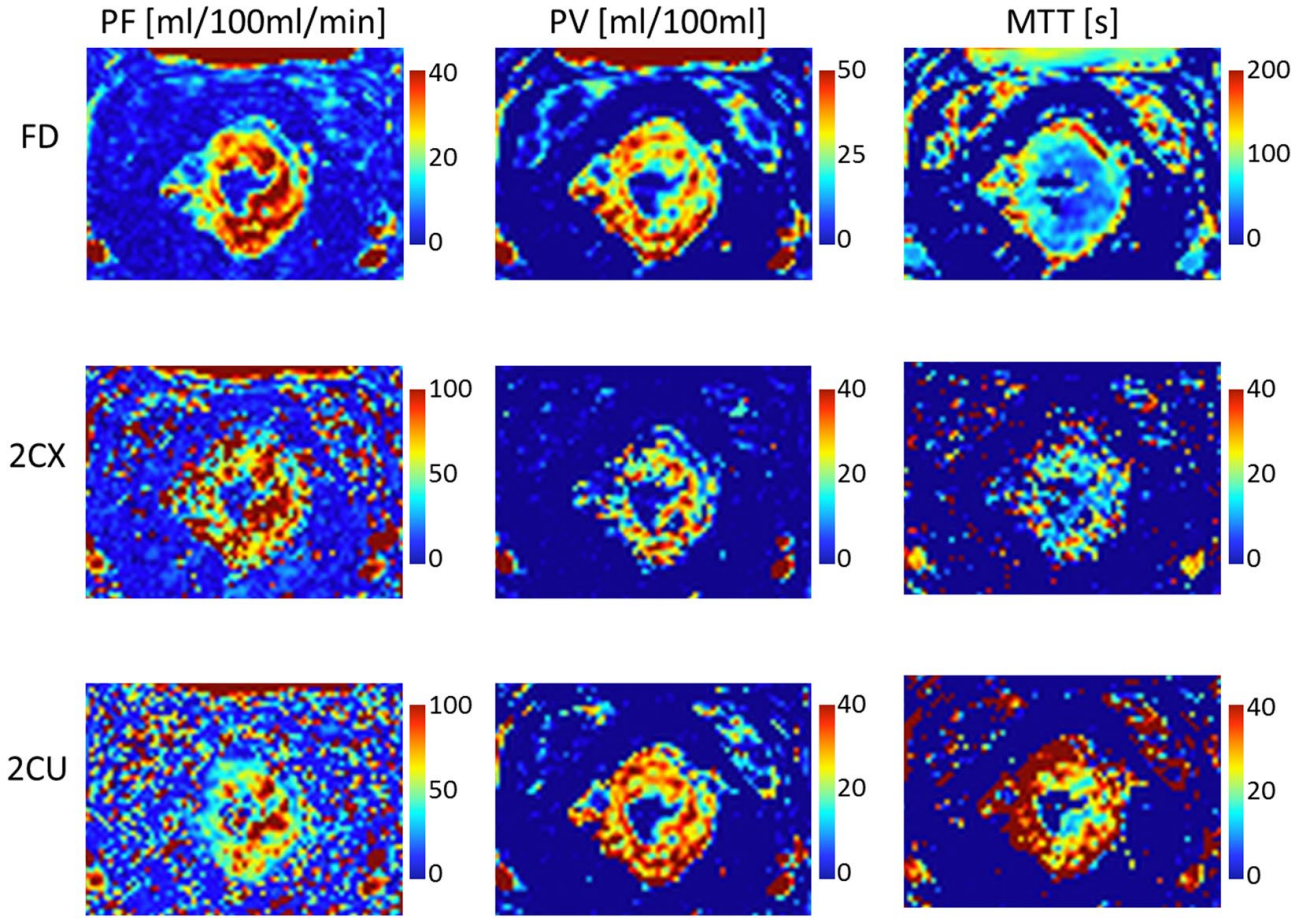
Pixel maps for PF, PV and MTT of the tumor region of one exemplarily chosen patient for the 2CU and 2CX compared to the deconvolution approach.

The time-concentration curves of the signal in the tumor tissue were acquired and the fit routine of both the 2CU and 2CX was performed with the scanned data points (Fig. 3). Pixel-wise fits of both pharmacokinetic models with the data in the region of the tumor volume were completed and parameter maps for PF, PV and MTT of the tumor region were generated. Maps of PF, PV and MTT for one exemplarily chosen patient are shown for the uptake and exchange model compared to the deconvolution approach in Fig. 4. Mean values for estimated parameters (PF_CX/CU/FD_ [ml/100 ml/min] = 68 ± 44/55 ± 36/36 ± 19, PV_CX/CU/FD_ [ml/100 ml] = 18 ± 11/22 ± 12/31 ± 15, MTT_CX/CU/FD_ [s] = 16 ± 9/25 ± 13/58 ± 16) and the goodness of fit measures (AIC_CX/CU_ = 169 ± 81/179 ± 77 and ${{\boldsymbol{\chi }}}^{2}$_CX/CU_ = 10 ± 12/9 ± 10) could be provided averaged over all 18 patients and additionally separated by cancer stage (Table 1).

**Table 1:** Overview of perfusion parameters and quality of fit parameters (stated in mean ± standard deviation) for the two compartment exchange and uptake model compared to the results of the fast deconvolution approach.

Perfusion Parameter - *Mean values of tumor tissue and (healthy tissue)*	2CX	2CU	FD
PF [ml/100 ml/min]	68 ± 44	55 ± 36	36 ± 19
	(37 ± 19)	(34 ± 16)	(27 ± 11)
PV [ml/100 ml]	18 ± 11	22 ± 12	31 ± 15
	(11 ± 4)	(11 ± 5)	(19 ± 7)
MTT [s]	16 ± 9	25 ± 13	58 ± 16
	(16 ± 5)	(20 ± 6)	(46 ± 16)
AIC	169 ± 81	179 ± 77	—
${{\boldsymbol{\chi }}}^{2}$	10 ± 12	9 ± 10	—
**T2 (3 patients)**			
PF [ml/100 ml/min]	41 ± 16	33 ± 5	34 ± 12
PV [ml/100 ml]	25 ± 9	31 ± 7	33 ± 6
MTT [s]	25 ± 13	38 ± 11	67 ± 18
**T3 (13 patients)**			
PF [ml/100 ml/min]	76 ± 47	61 ± 38	37 ± 21
PV [ml/100 ml]	16 ± 11	20 ± 12	31 ± 17
MTT [s]	13 ± 6	22 ± 11	55 ± 15
**T4 (1 patient)**			
PF [ml/100 ml/min]	34	33	27
PV [ml/100 ml]	17	17	26
MTT [s]	27	29	59

Values for healthy rectum wall are given in brackets and separate values for the different cancer stages are listed. Quality of fit parameters is not depicted for the deconvolution approach as values are calculated directly with this approach and no fit is required.

Both the 2CU and the 2CX show similar results for the mean values. However slight differences in the fit lead to higher values for PF (19%) for the 2CX whereas the mean values for the 2CU are higher for PV (18%) and MTT (36%). When comparing T2 and T4 with T3 results, it can be pointed out that all deconvolution values lie within the one sigma confidence interval. For 2CX and 2CU, values for MTT lie outside the one sigma confidence interval. Mean perfusion parameters for healthy rectum tissue showed smaller values for all three perfusion parameters (PF, PV, MTT) for FD and 2CU. For 2CX, PF und PV are smaller, MTT is constant.

**Figure 5:**
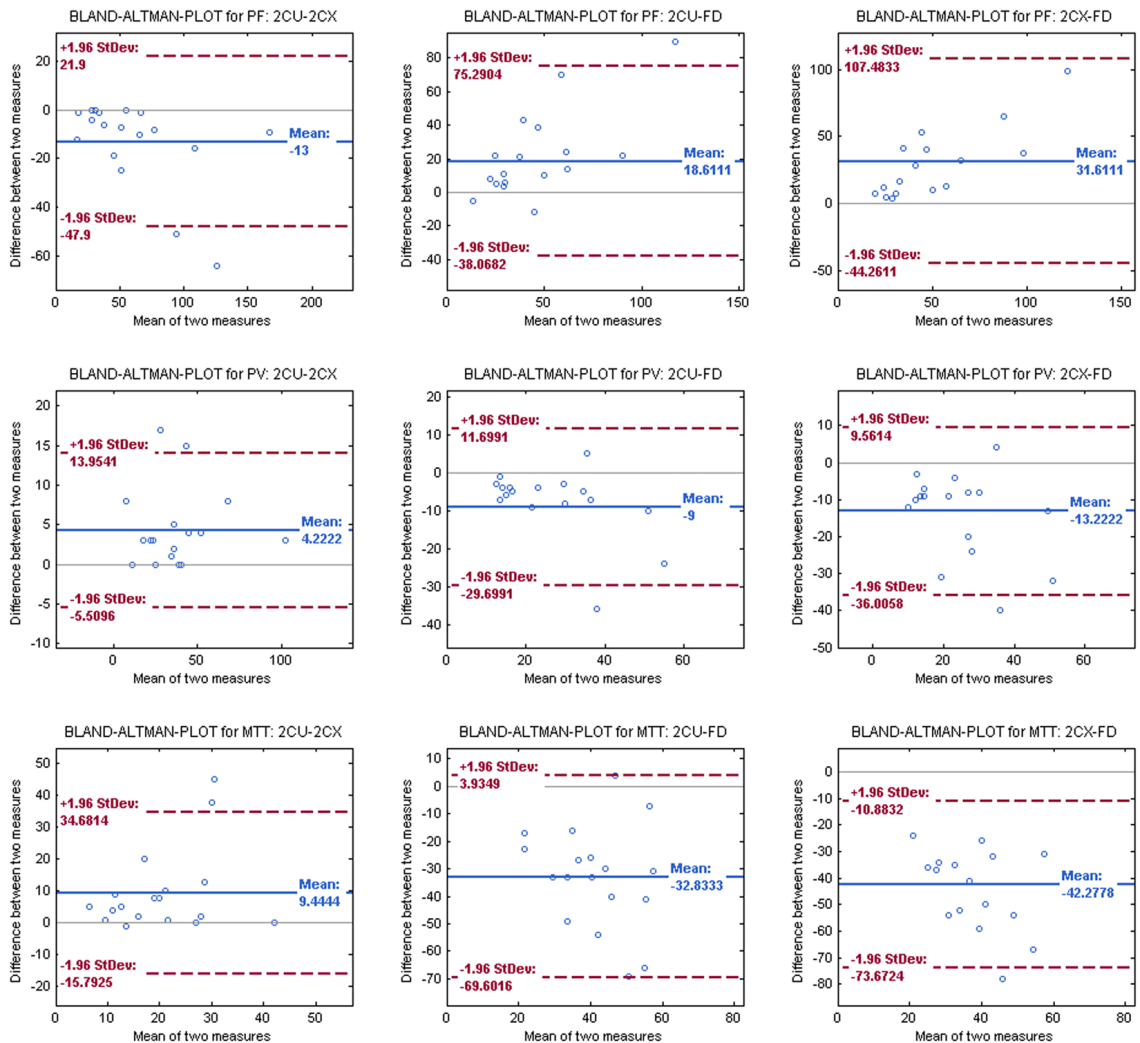
Bland-Altman plots of the perfusion parameters PF, PV and MTT of the applied models. Mean value of the difference between two measures is depicted in blue and band of agreement (placed at ± 1.96 standard deviations) depicted in red.

Parameters of the fit quality were calculated for both compartment models. These differed by 5% regarding AIC and 10% regarding ${{\boldsymbol{\chi }}}^{2}$. Compared to the compartment models (2CX/2CU), a distinct underestimation of the plasma flow (47%/35%) and an overestimation of mean transit time (72%/57%) and plasma volume (41%/29%) can be observed for the fast deconvolution approach. This is also visualized in the Bland-Altman plots in Fig. 5. In all plots, almost all data points are distributed within the band of agreement (placed at ± 1.96 standard deviations). A maximum of two outliers outside the band of agreement can be observed. Differences between two measures and corresponding standard deviations in the Bland-Altman plot of the fast deconvolution approach and one of the two-compartment models are generally higher than the comparison of the compartment models among each other.

### Statistical Analysis

Statistical analysis with the nonparametric paired Wilcoxon sign rank test showed significant differences for the comparison of all models. The p-values were smaller than 0.01 for all compared models and perfusion parameters (Table 2). Parameters for fit quality also showed significant differences, however with higher p values of 0.02 (AIC) and 0.04 (${{\boldsymbol{\chi }}}^{2}$).

**Table 2:** Statistical determination of p-values with Wilcoxon sign rank test of the comparison of perfusion parameters.

	FD - 2CX	FD - 2CU	2CX - 2CU
p (PF)	0.0012	0.0079	6.13E-05
p (PV)	3.51E-04	7.27E-04	2.44E-04
p (MTT)	1.96E-04	2.32E-04	6.34E-04
p (AIC)	—	—	0.02
p ${\boldsymbol{(}}{{\boldsymbol{\chi }}}^{2})$	—	—	0.04

P < 0.05 was considered as significantly different.

## Discussion

Several studies showed that the determination of perfusion parameters can serve as an additional tool for diagnosing and staging pathologic changes of organs and subsequently finding an appropriate therapy method^8,25^. A variety of models for the calculation of these parameters is available but there is no consensus which model represents the parameters best. Thus, it is important to use the most accurate model for determination of robust functional parameters to help establish their additional value for diagnostic imaging, here regarding rectal cancer. In this study, we investigated three different tracer kinetic approaches, namely the model-free fast deconvolution approach, the 2CU and the 2CX model, on its impact on the quantitative perfusion parameters for rectal cancer. To the best of our knowledge, this is the first paper examining compartmental model approaches in rectal cancer in contrast to previous papers dealing mainly with the deconvolution approach^25^ or the Tofts model^9^.

Mean values of the calculated quantitative perfusion parameters (PF, PV, MTT) show that they depend on a specific evaluation approach and are influenced by the tracer kinetic model. The Bland-Altman plots show that perfusion values in patients have a higher discrepancy and standard deviation when comparing the deconvolution approach with one of the compartment models as opposed to when comparing the two compartment models against each other. These results are in accordance with findings by Sourbron and Buckley^26^, where an underestimation of MTT and PV is described for the model-free analysis. This underestimation can occur due to long interstitial transit times of extravascular tracers. It is associated with an underestimation of the area under the impulse response function caused by too short acquisition times. They also reported a possible underestimation of PV for the extended Tofts model, whereas this effect does not occur for the compartment models^10^. As the uptake model is a simplification of the exchange model for specific cases, both models should yield similar results in these cases, whereas outliers would occur only when exactly one of the models fits the patient’s perfusion data. This is supported by the fact that, on the one hand, more outliers are detected for the comparison of the two compartment models, but on the other hand, standard deviation is smaller in contrast to the comparison with the deconvolution approach. The significant differences of the AIC and ${{\boldsymbol{\chi }}}^{2}$ values as a measure of the goodness of fit may be described by a few single outliers, as mean values show similar results. As most patients are grouped in T3 cancer stage, for a conclusion on whether the tumor stage is correlated with the perfusion parameters, the number of patients is too small. With a larger number of cases for all cancer stages, investigation of this context depicts an interesting future work.

A comparison with previously published papers investigating DCE-MRI of rectal cancer is challenging as a majority of studies used only the Tofts model for evaluation where the perfusion parameters obtained here are not calculated^8,9^. The two compartment models considered here have not been employed for the calculation of perfusion parameters in MR images before. However, studies using CT perfusion data show blood flow values between 60 and 110 ml/100 ml/min which corresponds to a plasma flow of 33 and 60.5 ml/100 ml/min, respectively, and is thus in a good agreement with the values we obtained in our study (cf. Table 1)^27,28^. Calculated perfusion values for healthy rectum tissue showed higher PF and PV for all models which is also in good agreement with^27^. However, due to the small size of the healthy rectum wall, only data of 10 patients was evaluated and the amount of pixels used was quite small. This could be an explanation why we measured higher (FD/2CU) or constant (2CX) MTT whereas in ref.^27^ it is reported to be shorter. Since the goodness of fit and perfusion parameters show reasonable results, it can be assumed that the two compartment models used are generally suitable for the quantification of perfusion in rectal cancer. The choice of the optimal model depends on several aspects, for instance, if only the perfusion has to be determined or if further information such as permeability and interstitial volume are to be studied. In ref.^12^ guidelines on selecting a tracer kinetic model are given. Based on these guidelines, it can be stated out that in some cases the 2CX and 2CU model provide advantages compared to the commonly used models. First of all, additional parameters can be useful for clinicians to describe the tumor and help to decide which therapy to use. Especially for antivascular drugs and a pre- and post-treatment observation, the deconvolution approach cannot give any information as it does not describe two compartments and the parameter K_trans_ of the Tofts model is not as specific as plasma flow and permeability surface area product in the 2CX model. Furthermore, the Tofts model assumes that the time taken for the contrast agent to pass through the plasma compartment (plasma MTT) is negligible. However, calculated mean transit times show, that this cannot be assumed and sign that the 2CX or 2CU model could describe the data better. Imaging quality is good enough to use a more complex model than model free deconvolution approach, which is probably more robust for images with poor data quality but does not describe the physiology properly^29,30^.

### Limitations

A general concern regarding DCE-MRI is a lack of standardization, concerning image acquisition routines and evaluation of obtained imaged data. The combination of different factors such as insufficient coverage of the entire tumor volume, varying type and amount of contrast media, arbitrary slice selection regarding AIF or tumor or the application of different model functions and methods for analysis complicates the comparison among different studies^31^. In this study, the choice of AIF and tumor slice followed the orientations used in previous studies and was strictly adhered to for all analyzed patients, guaranteeing best possible reproducibility^32^. Further improvement could be achieved by whole tumor analysis, accomplished by using the applied 3D sequences for tumor volume rendering, which would account for heterogeneous tumor tissue.

A limitation of our study is that no gold standard method for measuring the perfusion parameters, e.g. scintigraphy or positron emission tomography was employed and compared to the models. However, we aimed at comparing the quantification methods to each other rather than producing absolute values. As outlined before, our obtained values are found to be in the same range compared to other CT studies. Additionally, we did not perform a conversion of signal intensities to contrast agent concentration curves, but assumed a linear relationship^24^. As the conversion from signal intensities to contrast agent concentration is performed before the application of the pharmacokinetic models, this can be regarded as a systematic error. It should have a similar effect on all methods and consequently it would not change the comparison presented in this work. Nevertheless, the influence of conversion of signal intensities to contrast agent concentration curves will be examined in future research.

During our study, no correction of motion artefacts was performed. Peristaltic movement in the abdominal region can lead to wave-like deviations in the obtained data (Fig. 3) and might have an influence on the results. However, as all methods were applied to the same DCE-MRI data sets, any occurring motion artefacts would have affected the results equally and were not considered a major cause of error. Image registration approaches aiming to reduce motion artefacts in DCE-MRI exams of rectal cancer should be considered in prospective studies.

The study used two different scanners to obtain the patient data. However, the exact magnetic field strength given for both scanners Magnetom Skyra and Magnetom Trio is B_0_ = 2.89 T. Furthermore, a study investigated ADC values at these two scanners previously, and came to the conclusion that nearly identical values could be obtained^33^. Together with the fact that all other parameters are comparable and when calculating perfusion parameters, the baseline signal is subtracted, we assumed that influence of the scanner difference is neglectable.

We found significantly differences between all three approaches. This indicated that there is not only one right model for the complete patient cohort, but some models seem to fit better to some specific patients. An investigation on whether a specific tumor condition or stage is decisive for the choice of the model sounds promising. However, the investigated patient cohort is limited with 18 patients. A larger number of experimental studies to show reproducibility and validity of the parameter values would be necessary for a general statement on clinical usefulness and weather one model should be prioritized over the other. Though, the potential of other, physiologically also appropriate models compared to the widely used ones and the strong dependency of quantitative perfusion parameters on the chosen model could be shown here.

## Conclusion

A comparison of three different approaches to determine quantitative perfusion parameters in rectal cancer was performed. The model-free fast deconvolution approach and the two compartment exchange, and as well as the uptake model, were compared based on same patient data and ROI selection. An assessment of perfusion parameters using these models could be conducted for all patients. Determined perfusion parameters differed significantly among the chosen kinetic models. Fit quality showed similar mean value but significant difference among the patients. All models seem feasible to provide quantitative perfusion parameters in rectal cancer according to the obtained parameters. Consequently, they should be considered as an equivalent alternative to the commonly used Tofts model. These findings demonstrate that the choice of the kinetic model may have a significant effect on the results of a quantitative perfusion analysis.
